# Transient Covalency in Molten Uranium(III) Chloride

**DOI:** 10.1021/jacs.4c05765

**Published:** 2024-07-23

**Authors:** Dmitry S. Maltsev, Darren M. Driscoll, Yuanpeng Zhang, Joerg C. Neuefeind, Benjamin Reinhart, Can Agca, Debmalya Ray, Phillip W. Halstenberg, Mina Aziziha, Juliano Schorne-Pinto, Theodore M. Besmann, Vyacheslav S. Bryantsev, Sheng Dai, Santanu Roy, Alexander S. Ivanov

**Affiliations:** † Chemical Sciences Division, Oak Ridge National Laboratory, Oak Ridge, Tennessee 37831, United States; ‡ Department of Chemistry, University of Tennessee, Knoxville, Tennessee 37996, United States; § Neutron Scattering Division, Oak Ridge National Laboratory, Oak Ridge, Tennessee 37831, United States; ∥ Advanced Photon Source, Argonne National Laboratory, Lemont, Illinois 60439, United States; ⊥ Mechanical Engineering Department, University of South Carolina, Columbia, South Carolina 29208, United States

## Abstract

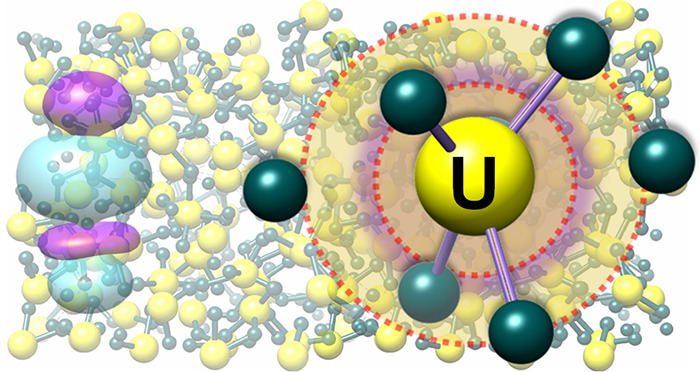

Uranium is arguably the most essential element in the actinide series, serving as a crucial component of nuclear fuels. While U is recognized for engaging the 5*f* orbitals in chemical bonds under normal conditions, little is known about its coordination chemistry and the nature of bonding interactions at extreme conditions of high temperature. Here we report experimental and computational evidence for the shrinkage of the average U–ligand distance in UCl_3_ upon the solid-to-molten phase transition, leading to the formation of a significant fraction of short, transient U–Cl bonds with the enhanced involvement of U 5*f* valence orbitals. These findings reveal that extreme temperatures create an unusual heterogeneous bonding environment around U(III) with distinct inner- and outer-coordination subshells.

The complex nature of chemical interactions, electronic structure, and redox properties of actinides presents deep challenges in science.^1−5^ Although prior studies in the solid state and aqueous solutions emphasize the importance of *f* electrons in actinide bonding,^6−10^ the interactions of actinides in the ionic environment at high temperatures are underresearched.^11,12^ This information is exceptionally challenging to obtain due to radioactivity and the extreme conditions of such experiments. In particular, understanding the chemistry of the molten uranium trichloride (UCl_3_) is crucial for the development of next-generation liquid nuclear fuel and handling radioactive waste.^13^ Yet, over the collection of previous experimental and computational works,^14−17^ there is still no clear consensus on the bond lengths and coordination structure of molten UCl_3_. The first structural studies using X-ray diffraction (XRD) at 1200 K coupled with classical molecular dynamics simulations showed that a fully ionic model failed to adequately describe the local UCl_3_ structure, and a better agreement was achieved by incorporating a degree of covalency into the U–Cl bond.^14^ Indeed, under normal conditions, uranium is known to provide its outermost 5*f* and 6*d* orbitals for mixing with ligand orbitals to afford some degree of covalency to the primarily ionic interactions.^9,18−21^ Thus, from fundamental and practical points of view, it is essential to study how the structure and bonding in UCl_3_ (Figure 1A) change upon melting.

**Figure 1:**
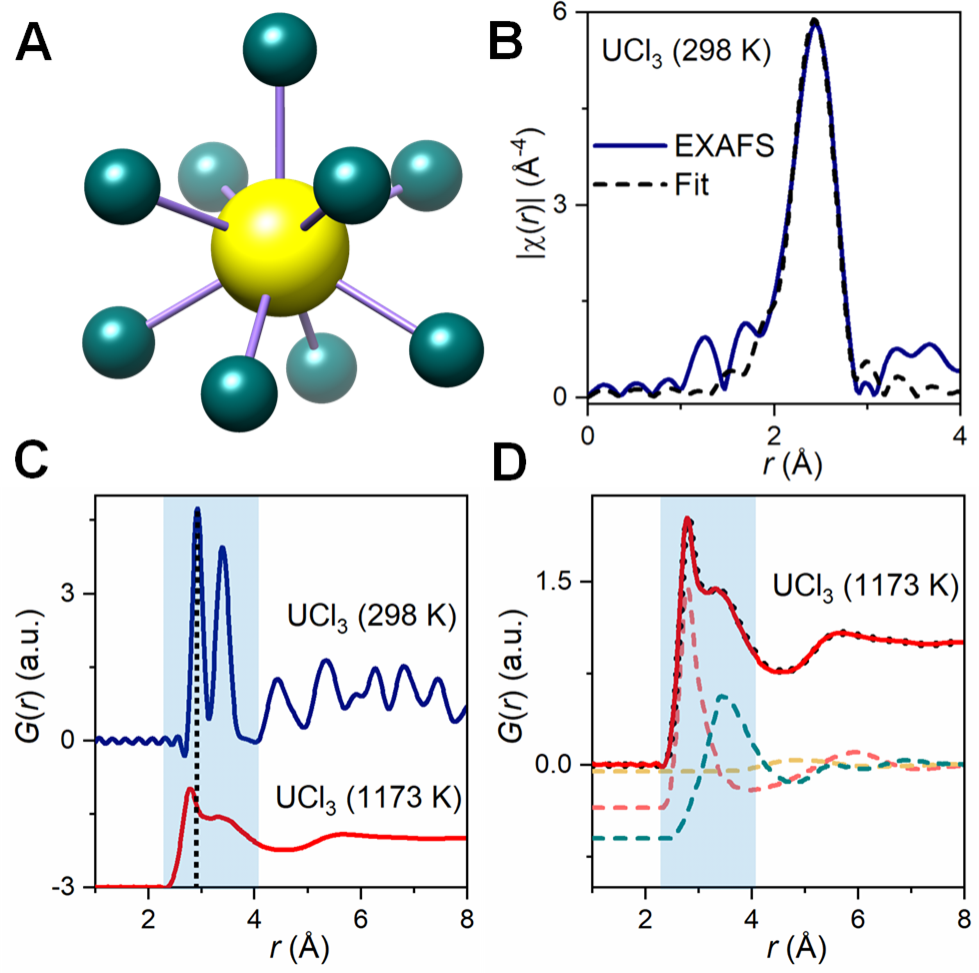
(A) Local structure of solid UCl_3_ and (B) the Fourier transform magnitude, |χ(*r*)|, of UCl_3_*L*_3_-edge EXAFS, *k*^3^χ. The spectrum is not phase-shift adjusted. (C) Neutron pair distribution functions, *G*(*r*), measured for solid (blue line) and molten (red line) UCl_3_, along with (D) the RMC fit for molten UCl_3_ (dashed line) and decomposition of *G*(*r*) into the U–Cl (pink), Cl–Cl (green), and U–U (yellow) ion pair correlations.

Toward this goal, we performed neutron scattering experiments and advanced *ab initio* molecular dynamics (AIMD) analyses to address the outstanding fundamental question of how the U–Cl bonding characteristics are affected by the extreme conditions of high temperature. Our results reveal the shrinkage of U–Cl bond lengths upon melting, leading to the formation of a highly heterogeneous coordination shell around U(III) ions. The AIMD and chemical bonding analyses in conjunction with the simulated Raman spectrum of molten UCl_3_ point to the presence of a large fraction of short transient U–Cl bonding interactions, which are spectroscopically active and exhibit a higher participation of U 5*f* orbitals than in the solid state.

According to single-crystal XRD, solid UCl_3_ adopts a tricapped trigonal prismatic configuration, where each U(III) center is surrounded by nine Cl atoms. Figure 1B shows our synchrotron extended X-ray absorption fine structure (EXAFS) spectroscopy results for the UCl_3_ sample, which was synthesized and handled under an inert atmosphere. The purity was confirmed by XRD, melting point measurements, and inductively coupled plasma optical emission spectrometry (Figures S1–S3, Table S1). Fitting the experimental EXAFS spectrum (Figure 1B, Figure S4) with a single U–Cl scattering path results in an average U–Cl distance of 2.909(9) Å (Debye–Waller factor, σ^2^ = 0.0079(11) Å^2^), consistent with the single-crystal XRD data (Table S2).

To study both local and extended structures, neutron scattering measurements were carried out on UCl_3_ powder that was contained in a quartz tube (Figure S5). The measured scattering intensities provide neutron structure factors, *S*(*Q*) (Figure S6), which were Fourier transformed to obtain pair distribution functions (PDFs), *G*(*r*), that are related to the probability of finding ion pairs with a given separation distance *r*. Figure 1C demonstrates significant changes in the PDF as UCl_3_ transitions from a solid (298 K) to a molten (1173 K) state. Broadening of *G*(*r*) features is observed upon heating with the complete loss of crystalline symmetries and long-range order correlations in the salt above the melting point (Figure 1C).

Based on the relative neutron weighting factors (Figure S7) and the UCl_3_ crystal structure, the first peak in *G*(*r*) is primarily dominated by the short-range U–Cl correlations, whereas the second peak is attributed to the Cl–Cl interactions. The features beyond 4 Å originate from the longer-range atom pair correlations. Interestingly, the first peak at 2.92 Å for solid UCl_3_ shifts toward a shorter distance upon melting, in direct contrast to thermal expansion expectations. This is likely associated with a decrease in the average coordination numbers on melting, often leading to shorter nearest-neighbor bond lengths.^22,23^ We note, however, that the intrinsically broad features in *G*(*r*) at 1173 K, associated with the liquid state, make quantitative interpretation of scattering results difficult. Thus, reverse Monte Carlo (RMC) modeling was performed to reproduce the experimental PDF of molten UCl_3_ and determine partial U–Cl, Cl–Cl, and U–U PDFs (Figure 1D). Our results show that U–Cl average bond length indeed shrinks on heating to the value of 2.78(1) Å in the molten state. A similar behavior was recently reported for molten tin at 530–1323 K, where the fraction of fluctuating short Sn–Sn covalent bonds unexpectedly increased, leading to a shift of the first peak in the PDF to a shorter distance at higher temperatures.^24^

To gain more insights into the observed U–Cl bond contraction phenomenon, we performed AIMD simulations. The theoretical *S*(*Q*) and *G*(*r*) were generated directly from the 60 ps AIMD trajectory and show very good agreement with the experimental data (Figures S6 and S8), validating our model. Key structural parameters align well with those determined by the PDF measurements and RMC fit, as can be judged from the analyses of radial distribution functions, *g*(*r*) (Figure S8). Figure 2A shows that the AIMD-predicted U–Cl bond length is 2.78 Å, shorter than the U–Cl bond length in the solid state (*r*_Solid_) and in good agreement with the RMC results. The actual boundary of the first Cl^–^ coordination shell around U(III) in the melt is where *g*(*r*) reaches the first minimum (*r* = *r*^†^ in Figure 2A). Within this first coordination shell, we define short U–Cl contacts in the inner subshell, for which *r* < *r*_Solid_, and long U–Cl interactions (*r*_Solid_ < *r* < *r*^†^) in the outer subshell. As one may see in Figure 2B, at any point of time, the former (∼55%) dominates the first coordination sphere, explaining the overall shrinkage of the average U–Cl bond seen in our PDF experiments. Interestingly, while these subshells are well preserved over time, the U(III) coordination number (CN) can rapidly deviate from its most-probable value of ∼7.5 (Figure S9), due to the high thermal energy.

**Figure 2:**
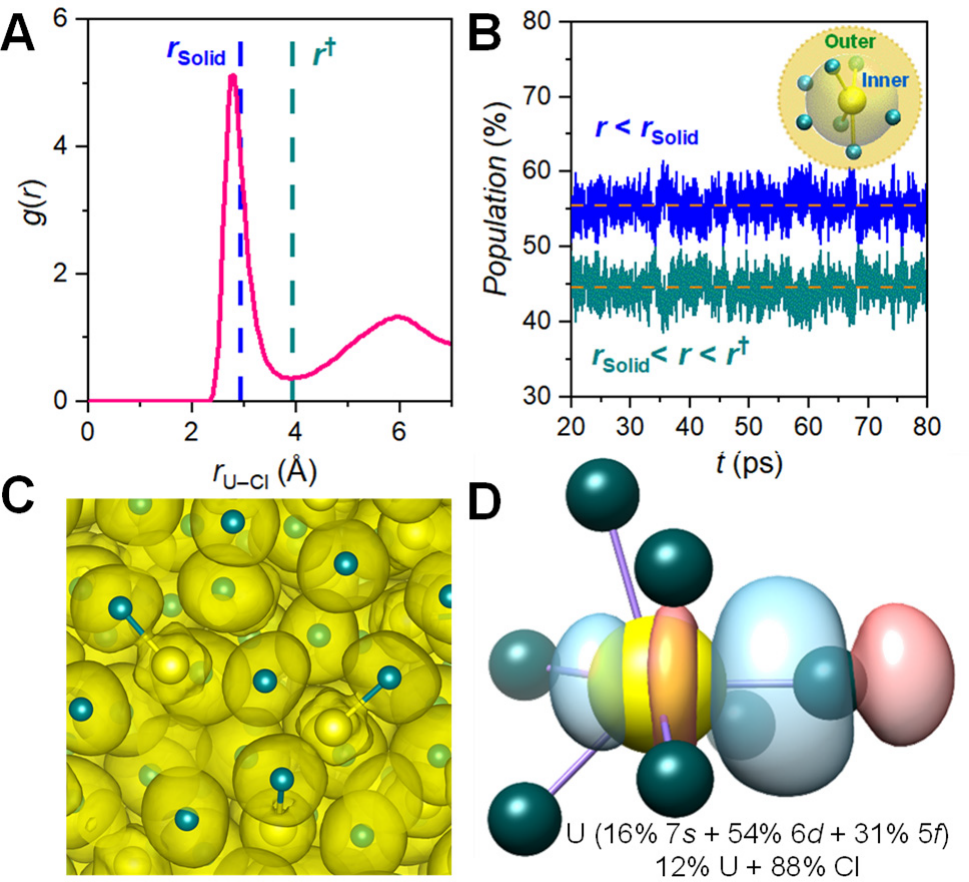
(A) U–Cl radial distribution function, *g*(*r*), highlighting the shorter U–Cl distance in the melt compared to the bond length (*r*_Solid_) in the solid state. *r*^†^ represents the boundary of the first coordination shell. (B) Time-dependent population of the inner (blue) and outer (teal) subshells. (C) Projection of the electron localization function for a snapshot from the UCl_3_ AIMD trajectory. (D) Selected short U–Cl σ-type dative bond NBO and its composition for the representative U(III) cluster in the melt.

The nature of the U–Cl chemical bonds in the two subshells was rigorously examined using quantum chemical methods, based on electron localization function,^25^ natural bond orbital (NBO),^26^ and quantum theory of atoms in molecules (QTAIM)^27^ analyses. Figure 2C shows that, in the inner subshell, the lone pairs of the chlorides are pointing toward U(III) with somewhat enhanced electron localization in the middle of the two atoms. In contrast, they lack directionality without being noticeably deformed in the outer subshell. This indicates the increased U participation in the short U–Cl bonds, whereas the longer U–Cl interactions are more of an ionic nature. The NBO analysis in Figure 2D for the representative cluster confirms the enhanced bonding in the inner subshell as a consequence of the high-temperature-induced shrinkage of the first coordination sphere around U(III), enabling better overlap of the Cl lone pairs with the U(III) acceptor orbitals. Although the U–Cl NBOs are strongly polarized toward Cl, our results point to the increased U(III) contribution and 5*f* orbital involvement in the inner subshell bonding at high temperatures as compared to the solid state (Table S3). Additionally, the enhanced bonding can be assessed employing Wiberg bond indices and QTAIM characteristics (Table S4), all showing the same trend of increased electron density sharing in the inner subshell U–Cl bonds of molten uranium trichloride. Furthermore, the projected density of states analysis shows that the overlap between U 5*f* and Cl 3*p* orbitals is stronger for the short U–Cl contacts as compared to the outer subshell interactions (Figure S10). This is consistent with our cluster model calculations and further confirms the presence of a slight orbital overlap between U and Cl, specifically at shorter distances. Thus, we anticipate that the role of the 5*f* valence orbitals of uranium in molten systems can be further explored using the Cl K-edge,^8^ U M_4,5_-edge high-energy-resolution X-ray absorption near edge structure and 3*d*4*f* resonant inelastic X-ray scattering spectroscopies^28−30^ in the future studies.

To gain insights into the dynamics of the U–Cl interactions dictating the stability of the two subshells, we determined the survival probability correlation function,^31^*C*(*t*), of the U-bound Cl for a range of cutoff distances, *r*_C_, corresponding to different boundaries of the subshells. Figure 3A shows that *C*(*t*) exhibits underdamped oscillations until ∼200 fs for the short U–Cl interactions (*r*_C_ < *r*_Solid_). However, this feature is absent for the longer U–Cl contacts (*r*_Solid_ < *r*_C_ < *r*^†^). The long-time behavior of all *C*(*t*)’s is the same as can be seen from the parallel exponential curves. Thus, a Cl^–^ ion spends ∼200 fs in the inner subshell before transitioning to the outer subshell, with the overall residence time in the first coordination sphere of ∼20 ps (obtained from the exponential fit to the long-time decay of the *C*(*t*) curve). This rather short lifetime of the U–Cl bonds at 1173 K points to their transient nature.

**Figure 3:**
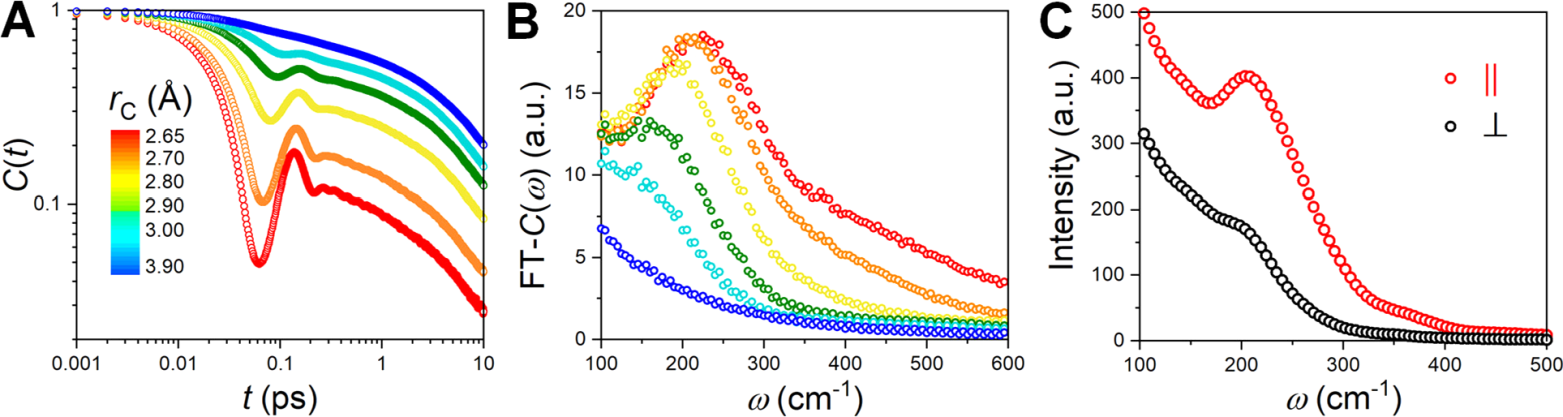
(A) Survival probability correlation functions, *C*(*t*), for a Cl^–^ bound U(III) plotted for various binding cutoff distances, *r*_C_, which are Fourier-transformed to obtain (B) FT-*C*(ω) spectra and compared with (C) simulated Raman spectra (parallel and perpendicular polarizations).

The important feature of covalent interactions is their directionality, usually giving rise to absorption bands in the infrared or Raman spectra.^9^ Our attempts to obtain experimental Raman results for the molten UCl_3_ were unsuccessful, due to the strong self-absorption by the sample.^32^ Nevertheless, to investigate whether the dynamic, metastable coordination environment of molten UCl_3_, especially the transient inner subshell, is spectroscopically resolvable, we computed Raman spectra from the AIMD trajectory using the Berry phase formalism.^33^ This method has previously shown very good reproducibility of the experimental data for various molten salt systems.^33,34^ Additionally, we obtained the Fourier-transformed spectra of *C*(*t*), FT-*C*(ω), for comparison. As depicted in Figure 3B and C, there is a striking match between FT-*C*(ω) for the inner subshell and the Raman spectrum with the parallel polarization. Both exhibit a well-resolved symmetric U–Cl stretch vibrational band at 200–250 cm^–1^ accompanied by a less noticeable high-frequency band at ∼350 cm^–1^. In contrast, FT-*C*(ω) for the outer subshell does not show any peaks. This is expected since the underdamped U–Cl oscillations are only present for the inner subshell, and thereby, only the short U–Cl contacts contribute to the distinct band. For the extended U–Cl bonds, their influence on the Raman spectra is analogous to the effect of the weakly complexing ions (e.g., alkali metal ions in molten salts), predominantly increasing the spectral intensity at the lower energy end without clearly differentiating into its own band. Thus, despite the transient nature, the inner subshell bonds are distinguishable from the outer subshell interactions and resolvable with vibrational spectroscopy.

Even the subtle yet important presence of 5*f* orbital covalency is frequently invoked to comprehend and rationalize the structure, reactivity, and spectroscopic properties of heavy element compounds.^35−42^ Recently, it has been demonstrated that high pressure can be utilized to modify the structure and bonding in actinide complexes.^43,44^ Our discovery of short transient bonds in molten UCl_3_, which show enhanced U 5*f* orbital participation and likely contributing to the highly heterogeneous coordination shell around U(III), illustrates that high temperature can also impact the fundamental characteristics of actinide compounds, including bond distances, coordination number, and local dynamics.^45^ These findings are expected to improve our fundamental understanding and prediction of the structurally diverse and dynamic coordination chemistry and speciation exhibited by actinides in molten phases.^11,12^

## Data Availability

Data sets for this article are made available within 30 days of the official acceptance date of this article by the journal in the Zenodo repository under the Digital Object Identifier (DOI): 10.5281/zenodo.12668490.

## Supporting Information Available

Synthesis and characterization of UCl_3_ and additional experimental and computational details, including neutron scattering measurements, chemical bonding, density of states, and coordination number analyses (PDF)

## Notes

This manuscript has been authored by UT-Battelle, LLC under Contract DE-AC05-00OR22725 with the U.S. Department of Energy. The United States Government retains and the publisher, by accepting the article for publication, acknowledges that the United States Government retains a nonexclusive, paid-up, irrevocable, worldwide license to publish or reproduce the published form of this manuscript, or allow others to do so, for United States Government purposes. The Department of Energy will provide public access to these results of federally sponsored research in accordance with the DOE Public Access Plan (http://energy.gov/downloads/doe-public-access-plan).
