# Supplementary material for: Transient Covalency in Molten Uranium(III) Chloride
Source: J Am Chem Soc. 2024 Jul 23;146(31):21220–4. doi: 10.1021/jacs.4c05765 (PMC11311222; doi:10.1021/jacs.4c05765)
Supplement: Supplementary file 1 — ja4c05765_si_001.pdf [file ja4c05765_si_001.pdf]

Supporting Information for

“Transient Covalency in Molten Uranium(III) Chloride”

Dmitry S. Maltsev,[†,ξ] Darren M. Driscoll,[†] Yuanpeng Zhang,[‡] Joerg C. Neuefeind,[‡] Benjamin Reinhart,[§] Can Agca,[†] Debmalya Ray,[†] Phillip W. Halstenberg,[†,ξ] Mina Aziziha,[¶] Juliano Schorne-Pinto,[¶] Theodore M. Besmann,[¶] Vyacheslav S. Bryantsev,[*,†] Sheng Dai,[†,ξ] Santanu Roy,[*,†] and Alexander S. Ivanov[*,†]

[†]Chemical Sciences Division, Oak Ridge National Laboratory, Oak Ridge, Tennessee 37831, United States

[ξ]Department of Chemistry, University of Tennessee, Knoxville, Tennessee 37996, United States

[‡]Neutron Scattering Division, Oak Ridge National Laboratory, Oak Ridge, Tennessee 37831, United States

[§]Advanced Photon Source, Argonne National Laboratory, Lemont, Illinois 60439, United States

[¶]Mechanical Engineering Department, University of South Carolina, Columbia, South Carolina 29208, United States

Contents

Supporting Methods 3
General considerations. 3
Synthesis of anhydrous uranium(III) chloride. 3
Uranium trichloride sample purity analyses.. 4
X-ray absorption spectroscopy experiments at the Advanced Photon Source (APS)................ 5
Neutron scattering experiments at the Spallation Neutron Source (SNS). 6
Ab Initio molecular dynamics (AIMD) simulations.. 7
Reverse Monte-Carlo (RMC) modeling.. 7
Chemical bonding analyses.. 8
U–Cl coordination number analysis... 8
Survival probability correlation function. 9
Table S1–S4…………………..………………………………………………………10-13
Figure S1–S10……………………………………………………………………………14-23
References.. 24

Supporting Methods

General considerations.

Attn: the natural uranium(III) chloride sample is radioactive and was shipped to APS and SNS facilities through corresponding radiation safety departments. Another potential hazard with the sample is related to the high temperature used during the neutron scattering measurement, which could result in severe burns if handled improperly. The chloride salts are relatively low hazard at room temperature. However, when the salts are melted at high temperature, they can interact with atmospheric moisture if exposed to room air to produce small amounts of HCl. This should only happen in the event of a cell rupture.

Synthesis of anhydrous uranium(III) chloride.

There are several approaches for synthesizing uranium chloride salts[1]. In this work, we used the reaction between uranium trioxide and hexachloropropene to form uranium tetrachloride, which was subsequently reduced to uranium(III) chloride employing zinc metal as a reducing agent. Uranyl nitrate salt (containing natural uranium U_{nat}: 99.28 wt% U^{238}, 0.71 wt% U^{235}, 0.01 wt% U^{234}) was taken as the initial compound in our adopted synthetic scheme. In the first stage, uranium peroxide was precipitated from uranium nitrate solution by the reaction with hydrogen peroxide (*Sigma-Aldrich, 30 wt.% in water*):

$$UO_2(NO_3)_2 + H_2O_2 + 2H_2O \rightarrow UO_4{\cdot}2H_2O + 2HNO_3 \qquad (1)$$

Obtained sediment was filtered, thoroughly washed, and then thermally decomposed in the air at 873-923 K to solid uranium trioxide:

$$UO_4{\cdot}2H_2O \rightarrow UO_3 + 2H_2O + \tfrac{1}{2}\,O_2 \qquad (2)$$

The obtained uranium trioxide was chlorinated by hexachloropropene (*Sigma-Aldrich, ≥ 90% purity*) at 433 K in a reactor with reverse coolant for 6 hours:

$$UO_3 + 3CCl_3CClCCl_2 \rightarrow UCl_4 + 3CCl_2CClCOCl + Cl_2 \qquad (3)$$

The sediment of uranium tetrachloride was carefully washed from the hexachloropropene excess and other reaction products using carbon tetrachloride and dried under vacuum.

The uranium trichloride was prepared using the metallothermic reduction method by zinc (*Sigma-Aldrich, 97.5 wt.% purity*) excess at 873 K in a quartz cell with a neck:

$$2UCl_4 + Zn_{(met)} \rightarrow 2UCl_3 + ZnCl_2 \qquad (4)$$

The cell was evacuated, filled with nitrogen, and placed in a preheated furnace. The unreacted zinc, uranium tetrachloride, and zinc chloride sublimed and condensed in the top cold part of the cell. The UCl_3 collected from the bottom part was carefully transferred and stored in a glovebox under an inert argon atmosphere.

Uranium trichloride sample purity analyses.

Sample handling: the UCl_3 salt was stored and handled in a MBraun Unilab Pro glovebox under high purity argon (Linde, > 99.999%), with total O_2 + H_2O concentrations kept below 5 ppm. The samples were weighed using an Ohaus PA84C scale (±0.1mg) inside the glovebox.

Composition analysis: three UCl_3 samples were digested and analyzed using inductively coupled plasma optical emission spectrometry (ICP-OES, Perkin-Elmer Avio 200) to measure impurity concentrations. A solution of 5% v/v of HNO_3 from the High-Purity Standards (HPS) Company was used as a blank for the ICP-OES as well as a solvent. The three samples which averaged 9.47 mg were each dissolved in 8 ml of 5% v/v of HNO_3, and digestion performed in a Teflon® (PTFE) vessel in a programable microwave digester (PerkinElmer's Titan MPS Microwave). The oven was ramped to 448 K and 20 bar in 30 min, and the samples held for 10 minutes and then cooled down to 323 K for removal from the digester. The trace element measurements utilized three different concentrations + blank (0, 0.5, 1, and 2 ppm) for calibration. The UCl_3 is considered 99.5% pure with respect to other cations (Table S1).

Structural analysis: crystallographic purity was confirmed by measuring XRD spectra in transmission mode. The samples were loaded into quartz capillaries with 0.01 mm thin walls and a diameter of 0.5 mm (Charles Supper) inside the glovebox and sealed with clay plugs to avoid exposure to air or moisture before removal to the diffractometer. Measurements were performed with a Rigaku 9kW Rotating Anode SmartLab X-ray Diffractometer with a Mo source ($K\alpha_1$ = 0.70926 Å and $K\alpha_2$ = 0.71359 Å radiation, zirconium filter). The XRD patterns were collected at room temperature over 5-50° 2θ with a step size of 0.01°, a counting time of 0.133 s, using a rotation rate of 3Hz. Rietveld refinement was performed using the Full Prof-Win Plot program[2, 3] A representative XRD pattern is provided in Figure S1. No additional peaks due to contaminants, such as those of hydrates or oxides, were observed, the sample being pure to the limit of the technique's sensitivity (2-3 wt.%). The obtained c/a ratio of 0.5809(1) is in excellent agreement with the values of Schleid *et al.*[4] (c/a = 0.5809(1)), and Vogel *et al.*[5] (c/a = 0.5800(1)).

Thermal analysis: heat-flux DSC measurements were carried out using a Netzsch-Gerätebau GmbH 404 F3 Pegasus® instrument in flowing 70 $mL{\cdot}min^{-1}$ of Ar (Linde, > 99.999%). Both heating and cooling thermal effects were analyzed to establish the melting and crystallization behavior of the salt. A sample of 19.1 mg was placed inside Ni liner and hermetically sealed in a high-pressure 100 µl chrome-nickel stainless-steel crucible as previously described.[6, 7] The instrument was calibrated using the a zero heating rate method of the IUPAC[8], which consists of heating the sample at different four heating rates varying overall by a factor of at least 10, which were 10, 5, 3, 1 $K{\cdot}min^{-1}$. The extrapolated peak onset temperatures are determined as a function heating rate and extrapolated linearly to a rate of 0 $K{\cdot}min^{-1}$. The accuracy for both heating and cooling was estimated to be within 3 K, and the error for the enthalpy of fusion on heating was less than 4%.

A melting point for the synthesized UCl_3 of 1117.9 ± 3 K was determined. Other measurements for UCl_3 were previously reported by Sooby *et al.*[9] (1115 ± 2 K), Kovacs *et al.*[10] (1115 ± 3 K), and Parker *et al.*[11] (1108 ± 0.1 K), and these values compare well with the determined melting point

for our sample. Some small impurity was observed in the 10 K·min^{-1} scan (inset in Figure S2).The measured enthalpy of fusion is 142.3 J/g (49.0 ± 2 kJ/mol^{-1}) (Figure S3) in excellent agreement with that of Kovacs *et al.*[10] (49.0 ± 2 kJ/mol^{-1}) and somewhat lower than that of Parker *et al.*[11] (52.0 ± 1 kJ/mol^{-1}).

Overall, it was estimated the purity was 98 ± 1 mol% due to the DSC small aberration at 10 K·min^{-1} run, with no additional phases identified by XRD, and ICP-OES only revealing trace concentrations of Ca, Fe, Mg, and Ni.

X-ray absorption spectroscopy experiments at the Advanced Photon Source (APS).

Uranium L_3-edge X-ray absorption spectroscopy (XAS) measurements were acquired at 12-BM of the Advance Photon Source. The synthesized UCl_3 powder was physically mixed with BN matrix and then sealed within a Kapton capillary (2.67 mm inner diameter). For transport to the beamline, the sample was placed into a quartz capillary, which was then flame-sealed, and doubly sealed within two epoxy-sealed Kapton capillaries all within an Ar glove box. XAS measurements were acquired at room temperature. The U spectrum was energy-aligned with respect to a Zr metal foil. XAS was acquired in florescence orientation using a seven-element Vortex detector.

Ejected photoelectrons are defined by their wavenumber (k) in relation to the absorption edge energy (E_0) through the equation:

$$k = \sqrt{2m_e(E - E_0)/\hbar^2} \qquad (5)$$

The experimental EXAFS oscillations of each sample, $\chi(k)$, are extracted from the normalized XAS data using subtraction of a spline and a cutoff distance (R_{BKG}) of 1.1 Å. For analysis of the EXAFS region, we use the EXAFS relationship given by:

$$\chi(k) = \sum_i \frac{F_i(k)S_0^2N_i}{kR_i^2} e^{-2k^2\sigma_i^2} e^{\frac{-2R_i}{\lambda(k)}} \sin(2kR_i + \delta_i(k) - \frac{4}{3}k^3C_{3,i}) \qquad (6)$$

where the index, i, is considered the path index and the $\chi(k)$ is calculated as the summation over all paths. For fitting of the EXAFS, FEFF6[12] was utilized with the experimental $\chi(k)$ data weighted by k^3 for all fits. In eq. 6, $F_i(k)$, $\delta_i(k)$, and $\lambda(k)$ represent the effective scattering amplitude, total phase shift, and mean-free-path of the photoelectron and each are derived from FEFF6. The degeneracy of the scattering path (N_i) was fixed to 9, based on the single-crystal XRD data. Therefore, the parameters still to be fit include, S_0^2, the many-body amplitude-reduction factor, R_i, the half-path length, σ^2_i, the Debye-Waller factor and $C_{3,i}$, the asymmetry of the distribution. Variation of the C_3 parameter was found to provide negligible changes to resulting EXAFS fits and was not varied in finally EXAFS fits. Additionally, a single nonstructural parameter for all paths, ΔE_0, is varied to align the $k = 0$ point of the experimental data and theory. Conventional EXAFS fitting focused exclusively on the first shell U–Cl scattering path, and the number of variables (4) stayed below the number of independent data points (6.9) available in the UCl_3 data with $k_{max} = 12.0$ Å^{-1}.

Neutron scattering experiments at the Spallation Neutron Source (SNS).

Sample preparation: the obtained uranium trichloride was crushed to fine powder in an argon glove box and added to a thin-walled NMR quartz tube (4.9635 ± 0.0065 mm O.D., 4.2065 ± 0.0065 mm I.D., wall thickness 0.38 mm; supplier: Wilmad-LabGlass). The quartz tube with UCl_3 was then flame sealed near the top under high vacuum (Figure S4). Before shipment to the beamline, the sample was tested for heating/cooling to the temperatures of the experiment (1173 K) to ensure there are no unwanted reactions or breaches.

Neutron total scattering experiments were performed using NOMAD beamline[13] at the Spallation Neutron Source (SNS), Oak Ridge National Laboratory. Flame-sealed quartz tube with UCl_3 was loaded into a vanadium sample can (Figure S4), and placed in the beamline furnace chamber, which was subsequently evacuated. A standard vacuum furnace was used to control the sample temperature at 1173 K. The total scattering structure factor, $S(Q)$, was obtained from the neutron time-of-flight data following standard data reduction protocols, including absolute normalization using a vanadium rod for reference and background subtraction. Scattering patterns obtained at room and temperatures above the melting point (1146 K) were collected for a total integrated proton charge of 4 C and 12 C, respectively.

The experimental structure function is given by:

$$S(Q) - 1 = \frac{I_{coh}(Q) - \sum_i x_i b_i^2}{[\sum_i x_i b_i]^2} \qquad (7)$$

where I_{coh} is coherent scattering intensity, x_i and b_i are the molar fraction and coherent neutron scattering length[14] of species i, respectively, and Q denotes the momentum transfer.

Real space pair distribution function (PDF), $G(r)$, was obtained by the Fourier transform of $S(Q)$:

$$G(r) - 1 = \frac{1}{2\pi^2 r \rho_0} \int_0^{Q_{max}} Q\,[S(Q) - 1]\sin(Qr)\,dQ \qquad (8)$$

where ρ_0 is the average number density of the system (number of atoms per unit volume). One can also define the partial subcomponents of $G(r)$ to provide information as to which pair interactions contribute to the overall PDF at specific distances:

$$G(r) - 1 = \sum_{ij} w_{ij}[g_{ij}(r) - 1] \qquad (9)$$

where w_{ij} is neutron weighting factor for an ionic pair factor defined as:

$$w_{ij} = \frac{(2-\delta_{ij})x_i x_j b_i b_j}{[\sum_i x_i b_i]^2} \qquad (10)$$

where δ_{ij} is one for $i=j$ and zero for $i\neq j$.

***Ab Initio* molecular dynamics (AIMD) simulations.**

We first performed classical polarizable ion model (PIM)-based[15, 16] nanosecond equilibrium molecular dynamics (MD) simulations for UCl_3 at 1173 K and 1 bar in the isothermal-isobaric ensembles (NPT). A snapshot with the experimental density[17] of UCl_3 from the equilibrated portion of the trajectory was chosen to generate the initial configuration for AIMD simulations (cubic box with length of 22.86979 Å, 96 U and 288 Cl, number density 0.032103 atoms·$Å^{-3}$, which corresponds to the UCl_3 experimental density of 4.589 g·cm^{-3} at 1173 K). These were performed using the PBE exchange-correlation functional[18-21] and Grimme's D3 dispersion correction[22] implemented through the Quickstep[23] module of the CP2K 6.1 package.[24, 25] The MOLOPT basis set[26] of double zeta valence plus polarization (DZVPMOLOPT) in conjunction with Goedecker-Teter-Hutter (GTH) pseudopotentials[27] were applied for all ions. Uranium was treated with a medium-core pseudopotential containing 78 electrons in core (Xe $4f^{14}$ $5d^{10}$) and the remaining 14 electrons ($6s^2$ $6p^6$ $7s^2$ $5f^3$ $6d^1$) in valence. The DFT+U method was employed for uranium ions with a partially filled 5f orbitals, using the Hubbard parameter U_{eff} = 2.00 eV recommended for CP2K.[24, 25] The DFT+U method for *f* elements is known to converge to multiple electronic states. With three alpha electrons in seven $5f$ orbitals there are 35 different orbital occupations. To guarantee that the lowest electronic state is found, we enforced a specific orbital occupation using the &ENFORCE_OCCUPATION section in CP2K. The occupation constrains were released after the first 20 SCF cycles. The lowest energy electronic state identified from all possible orbital occupations was used in the subsequent AIMD simulations. The orbital transformation method was employed with a FULL_ALL preconditioner and a conjugate gradient minimizer to achieve and accelerate the SCF convergence. A Nose-Hoover chain thermostat[28] with a velocity rescaling time constant of 1.0 picoseconds (ps) was used for the temperature coupling. A trajectory of 100 ps in length at 1173 K was generated using a 1.0 fs timestep. The last 60 ps were used for structural analysis. The total neutron $S(Q)$ and $G(r)$ were calculated from the AIMD simulation trajectory using the software package debyer.[29] For the Raman spectrum, we used the 40–70 ps trajectory interval for our simulations.

Reverse Monte-Carlo (RMC) modeling.

The neutron total scattering pattern of molten UCl_3 (1173 K) was fitted through the reverse Monte-Carlo (RMC) approach with the AIMD cell expanded to a 4 × 4 × 4 supercell, targeting an optimal match with experimental data in a metropolis manner. The RMCProfile package[30, 31] with such an algorithm implemented was employed to fit the neutron total scattering data in both real and reciprocal space. A total of 24576 ions (6144 U and 18432 Cl) in a cubic box with length of 91.479160 Å was used for the RMC fitting, with ~500 moves per ion generated and ~60 moves per ions accepted, overall. The minimum distance for each ionic pair was used as constraint to allow the system to fully relax. To guarantee the generality and good statistics of the obtained RMC results, the fit was performed using 15 different configurations and an average was taken over all RMC runs to obtain partial pair distribution functions. Hence, the resulting U-Cl distance in the molten state was reported considering a standard deviation: 2.78 ± 0.01 Å.

Chemical bonding analyses.

We used CP2K to produce the electron localization function (ELF)[32, 33] cube file from the AIMD trajectory snapshot. Ground-state single-point density functional theory (DFT) calculations (PBE0-D3 level of theory)[34] were performed using the Gaussian 16, Revision A.03 program package[35] on the representative UCl_9^{6-} and UCl_8^{5-} clusters based on the experimental geometry (solid state) or AIMD snapshot structure (molten state). This enlisted unrestricted Kohn–Sham methods, with the aug-cc-pVDZ basis set[36] for the chloride atoms. The Stuttgart small-core (SSC) potential to account for relativistic effects and the associated contracted basis set[37] was used for U, and the cluster was treated as a negatively charged quartet with three unpaired *f*-electrons. The bonding in UCl_3 and Wiberg bond indices (WBIs) were examined by using the natural bond orbital (NBO) methodology[38], as implemented in the NBO7 program.[39, 40] We further analyzed the electron densities at bond critical points (BCPs) for each U–Cl bond in the representative clusters at molten and solid states using the quantum theory of atoms in molecules (QTAIM) approach[41], which is based on topological features of the electron density. The QTAIM analysis was performed using the Multiwfn program.[42] The electron densities at BCPs for each U–Cl bond are summarized in Table S4. Molecular orbital diagrams were drawn with an isovalue of 0.02 a.u. Model representations in the figures were prepared using the UCSF Chimera software.[43] For the density of states (DOS) analysis, we performed spin-polarized (ferromagnetic state) single point calculations using the VASP 6 software[44, 45] on the snapshot configuration from UCl_3 AIMD trajectory. The calculations were done using PBE+U DFT functional.[46] Hubbard U value of 4 eV was used for U 5*f* electrons. A planewave basis set of 400 eV and standard PAW pseudopotentials were employed. Due to the large unit cell the Brillouin zone was sampled using the Gamma point approximation.

U–Cl coordination number analysis.

Considering r_i as the distance between the ith Cl^- and a U(III) ion and $r^\dagger$ as the location of the boundary of the first chloride coordination shell, the coordination number (CN) is defined using a smooth function, *f*:

$$CN = \sum_{i=1}^{N_{\text{Cl}}} \frac{1-\left(\frac{r_i}{r^\dagger}\right)^{12}}{1-\left(\frac{r_i}{r^\dagger}\right)^{24}} = \sum_{i=1}^{N_{\text{Cl}}} f_i^{\text{Cl}} \qquad (11)$$

$r^\dagger$ is obtained from the first minimum of the U–Cl *g(r)*, which appears after the first peak. N_{Cl} is the total number of chloride ions. The function, *f*, allows smooth transitions of Cl^- ions across the boundary. The powers, (12, 24), ensure that both the smoothness and correctness of CN are maintained as discussed in our earlier studies.[47] We compute the free energy profile of CN from its probability distribution, P(CN), i.e., $W(\text{CN}) = -k_B T \ln[P(\text{CN})]$, where T is the temperature and k_B in the Boltzmann constant.

Survival probability correlation function.

The survival probability correlation function, $C(t)$, is defined as[48]:

$$C(t)=\langle P_i(t,t+\delta t,)\rangle_{i,t}/\langle P(t,t)\rangle_{i,t}. \quad (12)$$

where, the survival probability, P, is assigned a value of 1 when a Cl^- is found in the first solvation shell of U(III) at both times t and $t+\delta t$. Otherwise, P is set to zero. $\langle\ldots\rangle_{i,t}$ indicates averaging over U–Cl pairs and time. We carried out the $C(t)$ calculations for different cutoff distances representing the boundary of the coordination shell (from short U–Cl bond distance to the actual cutoff distance obtained from the U–Cl $g(r)$), and Fourier-transformed $C(t)$ to resolve the vibrational signatures of the coordination shell. The expression for the Fourier transformed spectra, FT-$C(\omega)$, is:

$$\mathrm{FT}-C(\omega)=\int_0^{t_{\max}} C(t)\cos(\omega t) \quad (13)$$

$t_{\max}$ is the maximum correlation time and ω is the frequency of U–Cl symmetric bond stretching vibration.

Table S1. The impurity metal concentrations, ppm (mg/1000g U), for the UCl_3 sample measured by ICP-OES.

Ca (ppm)	Fe (ppm)	Mg (ppm)	Ni (ppm)
438(60)	36(4)	223(43)	65(4)

Table S2. Comparsion of UCl_3 crystallographic data (coordination number (CN) and average U–Cl bond length from the available crystal structures identified by their respective Cambridge Crystallographic Data Centre (CCDC) number) and our EXAFS fitting parameters derived from the UCl_3 sample at room temperature. 1σ errors in U–Cl bond distance are computed from the covariance matrix of the non-linear minimization of the EXAFS fit.[12]

	EXAFS	CCDC 1592256	CCDC 1603035	CCDC 1604970	CCDC 1605586	CCDC 1706916
CN	9*	9	9	9	9	9
r (U–Cl), Å	2.909(9)	2.933	2.959	2.928	2.965	2.931
σ^2 (U–Cl), Å^2	0.0079(11)	-	-	-	-	-
L_3 ΔE_0, eV/S_0^2	3(1)/0.61(8)	-	-	-	-	-

CN, coordination number; S_0^2, amplitude-reduction factor; *r*, interatomic distance; σ^2, Debye-Waller factor; ΔE_0, a single non-structural parameter for all paths, was varied to align the *k*=0 point of the experimental data and theory. *Fixed parameters. *k* - window: 2.7 – 12.0 Å^{-1}; *r* - window: 2.0 – 3.2 Å were used for the EXAFS fit.

Table S3. Comparison of the selected short U–Cl bond NBO for the molten state and U–Cl bond NBO for UCl_3 crystal structure. The main difference is found in the increased U(III) contribution at the high temperature (molten state, inner sub-shell), as well as the U 5*f* orbital involvement in the chemical bonds.

	Percentage of the NBO on each natural atomic hybrid		Composition of U natural atomic hybrid		
	U	Cl	7*s*	6*d*	5*f*
Molten state (1173K, inner sub-shell) U–Cl	12%	88%	16%	54%	31%
Solid state, crystal U–Cl	10%	90%	23%	73%	14%

Table S4: Wiberg bond indices (WBIs) and computed electron densities (eV/Å^3) and -G/V values for U-Cl bonds in the representative UCl_8^{5-} (molten state) and UCl_9^{6-} (solid state) clusters using QTAIM analysis. The obtained WBIs exhibit higher values for the short bonds and the QTAIM analysis shows an average electron density of ~0.08 eV/ Å^3 and -G/V (ratio of kinetic and potential energy) of 0.75 for the U-Cl bond lengths that are shorter than 2.6 Å. We note that QTAIM analysis is known to be not sufficiently sensitive to minor changes in actinide 5*f* bonding.[49] However, our QTAIM results indicate that unlike the long ionic bonds in the outer sub-shell, the short predominantly ionic U-Cl bonds in the inner sub-shell are indeed accompanied by some covalency.

UCl_8^{5-} (molten state)				
Index	Bond Length (Å)	Wiberg Bond Indices	Electron Density (eV/Å^3)	-G/V
1	2.533	1.212	0.083	0.73
2	2.555	1.202	0.081	0.73
3	2.616	1.119	0.071	0.76
4	2.923	0.795	0.039	0.85
5	2.969	0.724	0.036	0.86
6	2.973	0.775	0.036	0.86
7	3.222	0.573	0.022	0.96
8	3.930	0.240	-	-
UCl_9^{6-} (solid state)				
Index	Bond Length (Å)	Wiberg Bond Indices	Electron Density (eV/Å^3)	-G/V
1	2.928	0.621	0.037	0.91
2	2.928	0.621	0.037	0.91
3	2.928	0.607	0.037	0.91
4	2.928	0.607	0.037	0.91
5	2.928	0.593	0.037	0.90
6	2.928	0.593	0.037	0.90
7	2.935	0.569	0.036	0.92
8	2.935	0.593	0.037	0.90
9	2.935	0.593	0.037	0.90

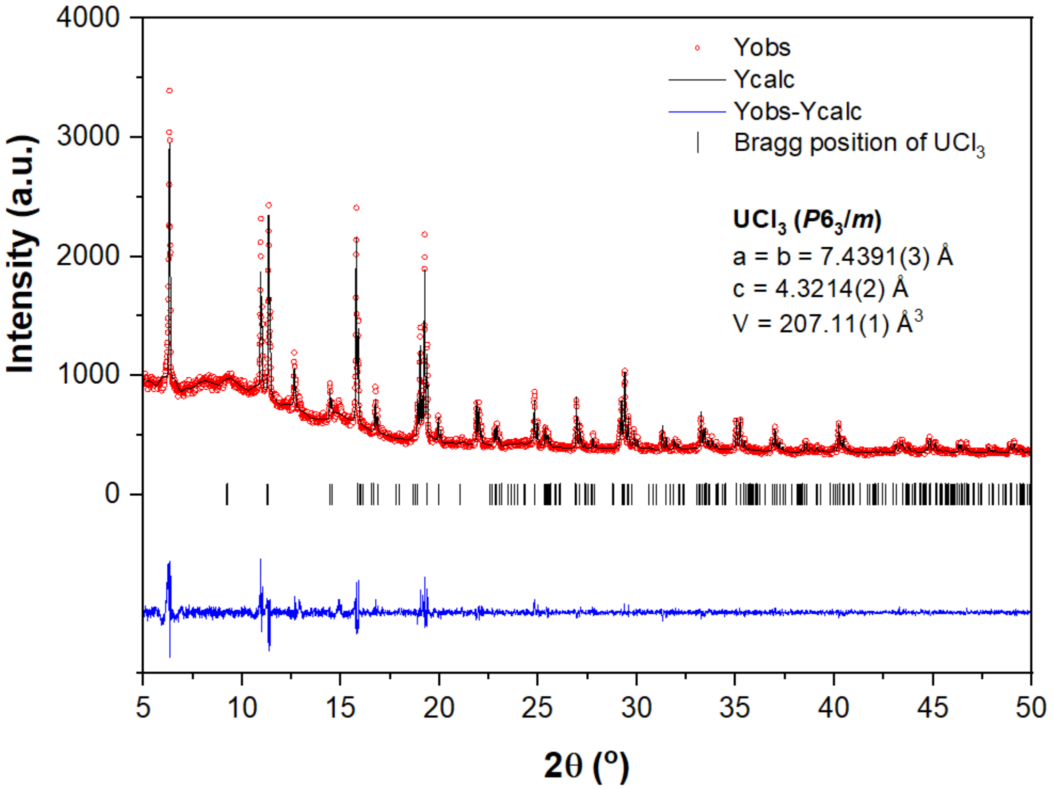

Figure S1. Room temperature refined powder transmission XRD pattern for UCl_3.

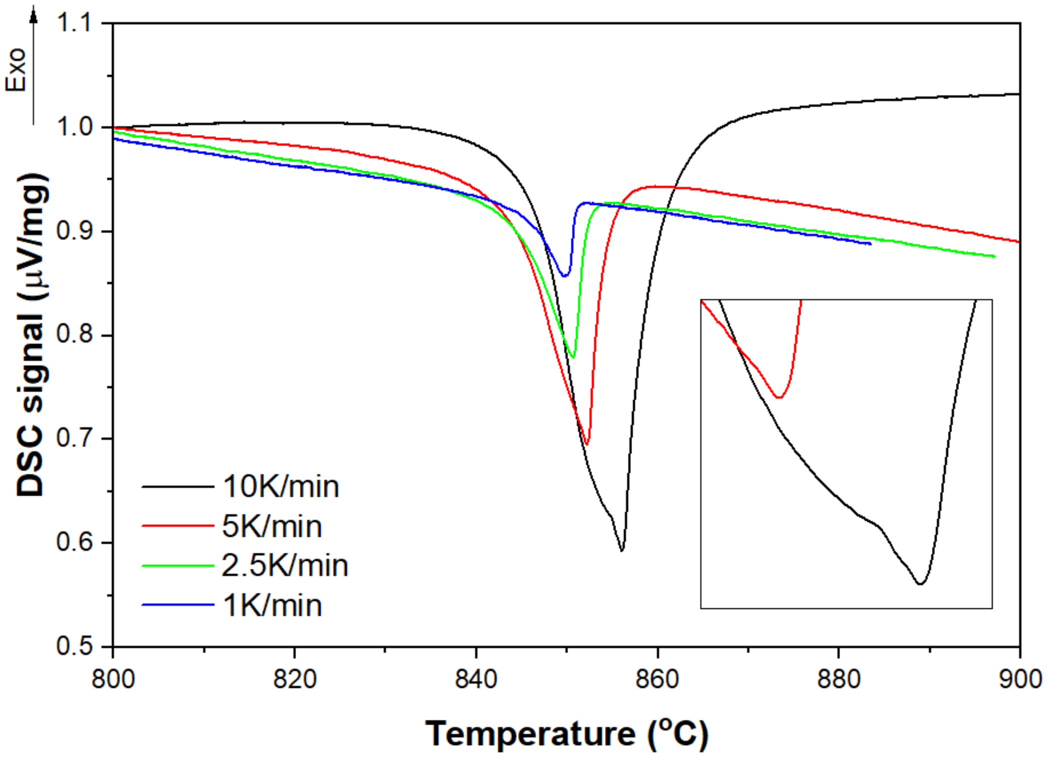

Figure S2. DSC curves for the UCl_3 sample on heating at the four rates of 1, 2.5, 5 and 10 K·min^{-1}. Inset is an expanded view of the peaks obtained at 5 K·min^{-1} and 10 K·min^{-1}.

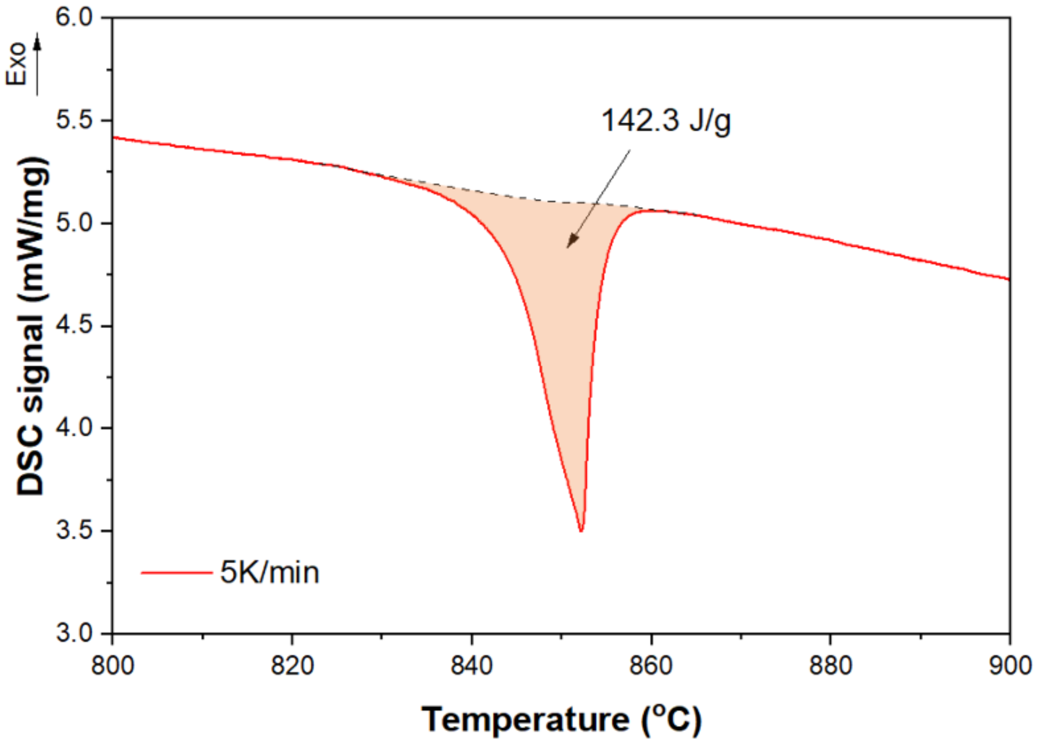

Figure S3. DSC scan at 5 K·min^{-1} used to obtain the enthalpy of fusion for UCl_3 sample measured.

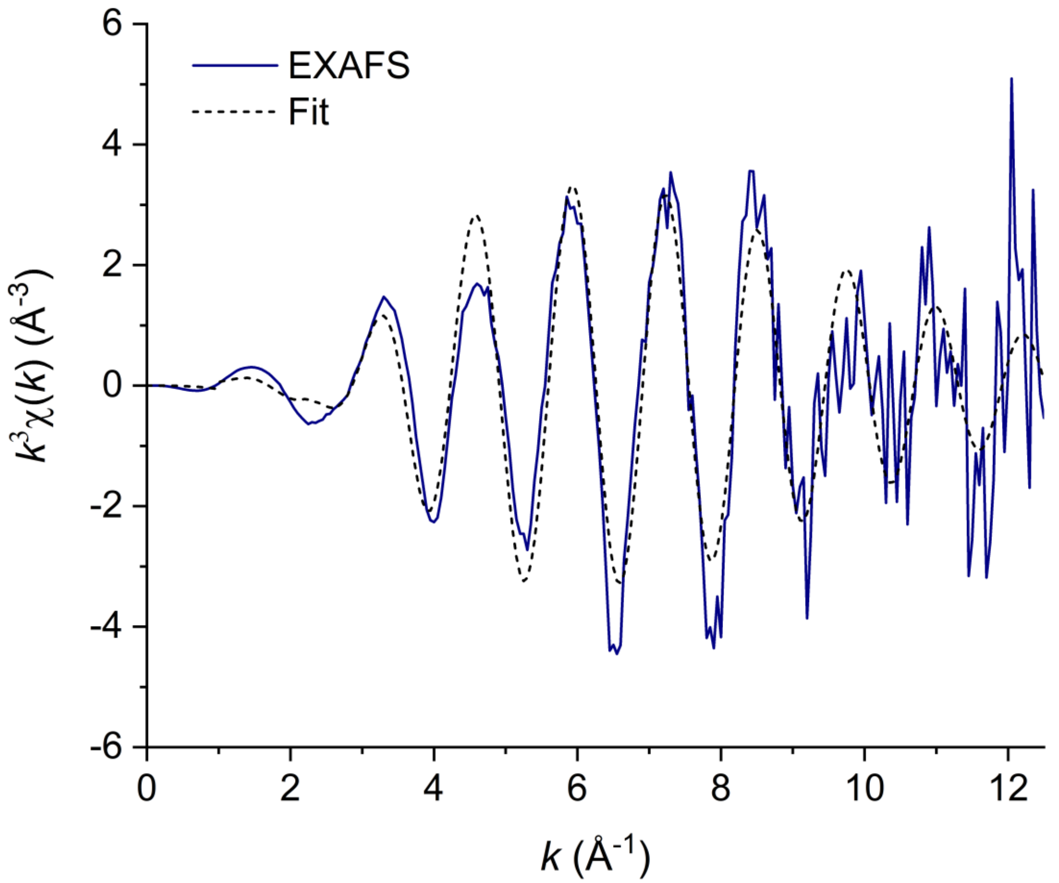

Figure S4. L_3-edge EXAFS spectrum of the synthesized UCl_3 salt at room temperature where k is the energy of the photoelectron in wavenumbers and $k^3\chi(k)$ is the k^3-weighted EXAFS function. Data between 2.7 and 12.0 Å^{-1} were Fourier transformed using a Hanning window to obtain real-space information presented in Figure 1A (main text).

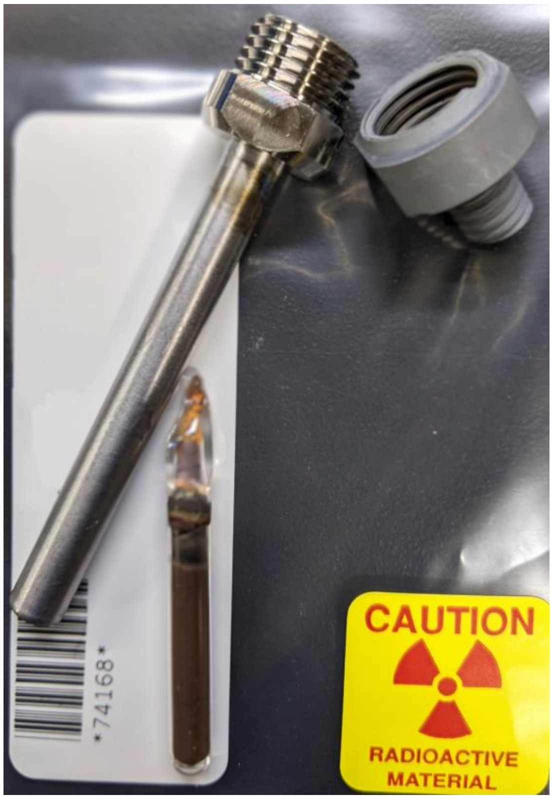

Figure S5. UCl_3 sample in a sealed quartz tube and vanadium containment used for neutron scattering experiments at NOMAD beamline.

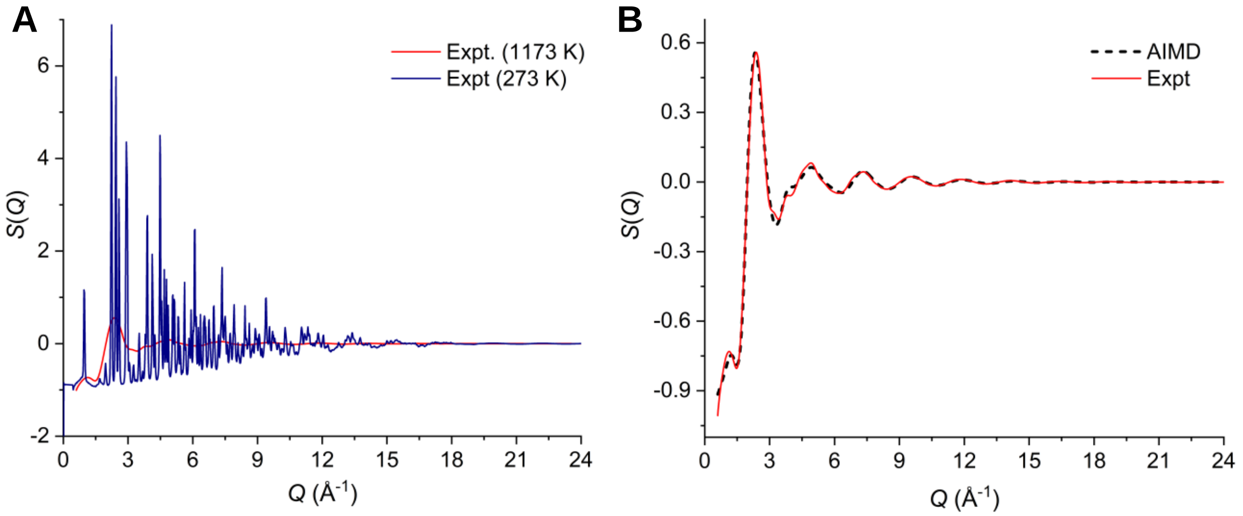

Figure S6. (A) Neutron structure factors, $S(Q)$s, obtained from neutron scattering measurements of UCl_3 at room temperature (blue) and 1173 K (red). (B) Comparison of $S(Q)$s for molten UCl_3 at 1173 K, obtained experimentally (solid red line) and using AIMD simulations (dashed black line).

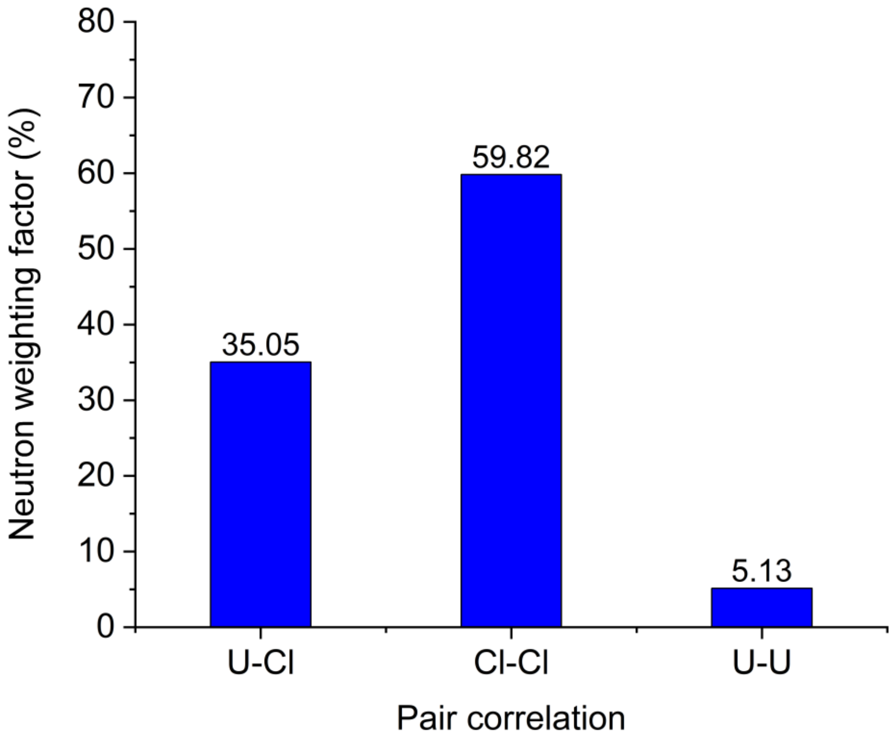

Figure S7. The relative neutron weighting factors for the pair correlations in the UCl_3 system. As one may see, most of the scattering comes from the chlorides and thus total neutron scattering patterns and the corresponding PDFs in Figure 1 (main text) are primarily dominated by U–Cl and Cl–Cl correlations, whereas the U–U contribution is difficult to discern from the total neutron PDFs.

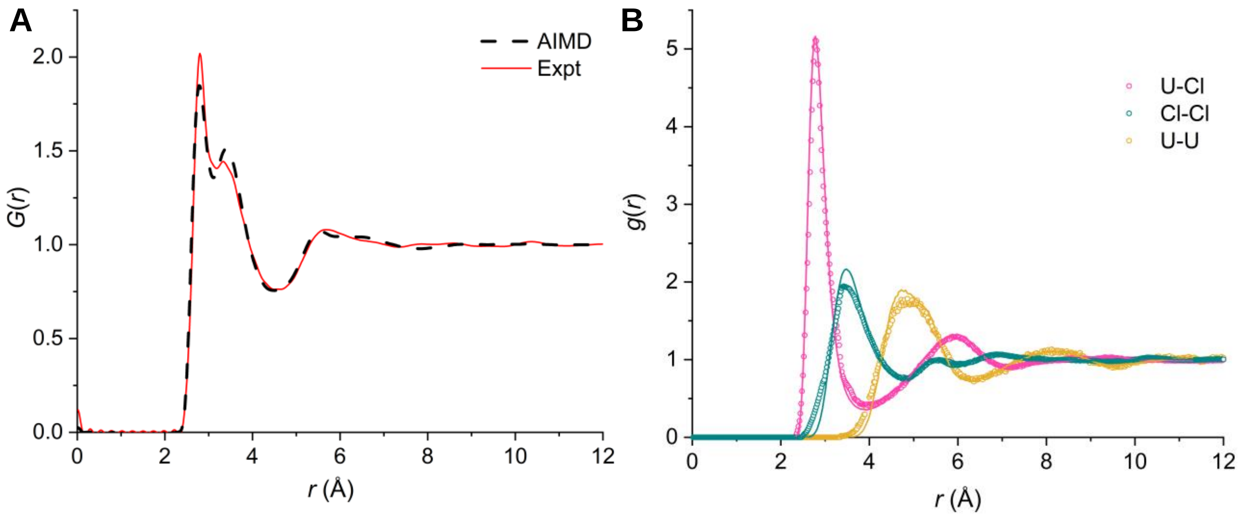

Figure S8. (A) Comparison of neutron pair distribution functions, $G(r)$s, for molten UCl_3 at 1173 K, obtained experimentally (solid red line) and using AIMD simulations (dashed black line). (B) Comparison of U-Cl (pink), Cl-Cl (green), and U-U (yellow) radial distribution functions, $g(r)$s, obtained from RMC fit (circles) and AIMD simulations (solid lines).

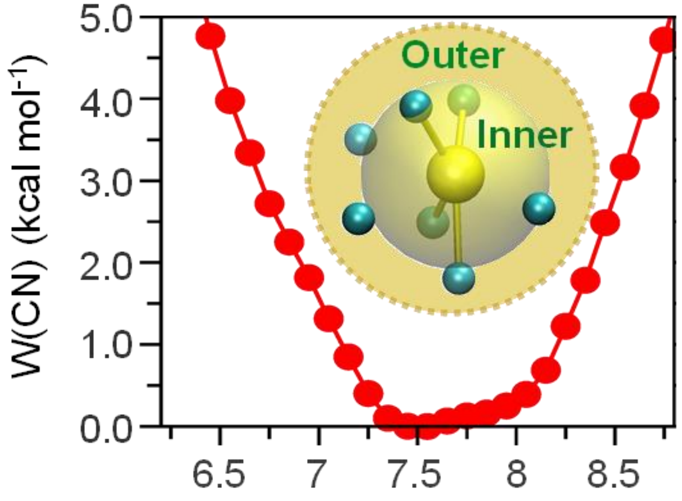

Figure S9. Free energy profile, W(CN), of Cl^- coordination number (CN) for U(III), along with a snapshot of the representative U(III) solvation shell.

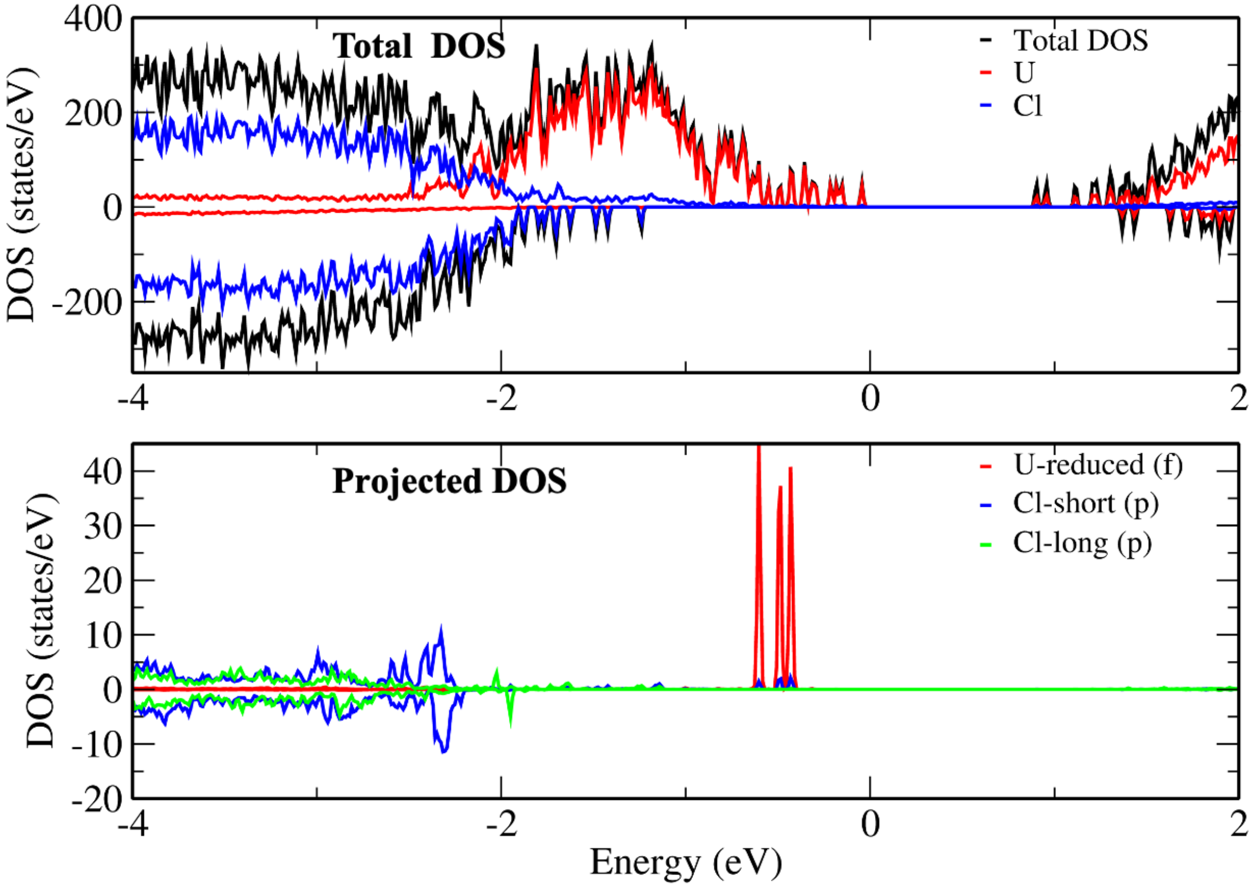

Figure S10. Total DOS and projected DOS of UCl_3 molten salt using PBE+U level of theory (top panel). Projected DOS of the most reduced U center and surrounding Cl using PBE+U level of theory (bottom panel).

References

(1) Brauer, G. Handbook of Preparative Inorganic Chemistry. Academic Press.: 1963; Vol. 4.

(2) Rodríguez-Carvajal, J. Recent Advances in Magnetic Structure Determination by Neutron Powder Diffraction. *Physica B: Condensed Matter* **1993**, *192* (1), 55-69.

(3) Rietveld, H. M. A Profile Refinement Method for Nuclear and Magnetic Structures. *Journal of Applied Crystallography* **1969**, *2* (2), 65-71.

(4) Schleid, T.; Meyer, G.; Morss, L. R. Facile Synthesis of UCl_4 and $ThCl_4$, Metallothermic Reductions of UCl_4 with Alkali Metals and Crystal Structure Refinements of UCl_3, UCl_4 and Cs_2UCl_6. *Journal of the Less Common Metals* **1987**, *132* (1), 69-77.

(5) Vogel, S. C.; Andersson, D. A.; Monreal, M. J.; Jackson, J. M.; Parker, S. S.; Wang, G.; Yang, P.; Zhang, J. Crystal Structure Evolution of UCl_3 from Room Temperature to Melting. *JOM* **2021**, *73* (11), 3555-3563.

(6) Piro, M. H. A.; Lipkina, K.; Hallatt, D. Exploring Crucible Designs for Differential Scanning Calorimetry Measurements of Fluoride Salts. *Thermochimica Acta* **2021**, *699*, 178860.

(7) Yingling, J. A.; Schorne-Pinto, J.; Aziziha, M.; Ard, J. C.; Mofrad, A. M.; Christian, M. S.; Dixon, C. M.; Besmann, T. M. Thermodynamic Measurements and Assessments for LiCl-NaCl-KCl-UCl_3 Systems. *The Journal of Chemical Thermodynamics* **2023**, *179*, 106974.

(8) Della Gatta, G.; Richardson, M. J.; Sarge, S. M.; Stølen, S. Standards, Calibration, and Guidelines in Microcalorimetry. Part 2. Calibration Standards for Differential Scanning Calorimetry (IUPAC Technical Report). **2006**, *78* (7), 1455-1476.

(9) Sooby, E. S.; Nelson, A. T.; White, J. T.; McIntyre, P. M. Measurements of the Liquidus Surface and Solidus Transitions of the NaCl–UCl_3 and NaCl–UCl_3–$CeCl_3$ Phase Diagrams. *J Nucl Mater* **2015**, *466*, 280-285.

(10) Kovács, A.; Booij, A. S.; Cordfunke, E. H. P.; Kok-Scheele, A.; Konings, R. J. M. On the Fusion and Vaporisation Behaviour of UCl_3. *Journal of Alloys and Compounds* **1996**, *241* (1), 95-97.

(11) Parker, S. S.; Long, A.; Lhermitte, C.; Vogel, S.; Monreal, M.; Jackson, J. M. Thermophysical Properties of Liquid Chlorides from 600 to 1600 K: Melt Point, Enthalpy of Fusion, and Volumetric Expansion. *Journal of Molecular Liquids* **2022**, *346*, 118147.

(12) Ravel, B.; Newville, M. ATHENA, ARTEMIS, HEPHAESTUS: Data Analysis for X-Ray Absorption Spectroscopy Using IFEFFIT. *Journal of Synchrotron Radiation* **2005**, *12* (4), 537-541.

(13) Neuefeind, J.; Feygenson, M.; Carruth, J.; Hoffmann, R.; Chipley, K. K. The Nanoscale Ordered MAterials Diffractometer NOMAD at the Spallation Neutron Source SNS. *Nuclear Instruments and Methods in Physics Research Section B: Beam Interactions with Materials and Atoms* **2012**, *287*, 68-75.

(14) Sears, V. F. Neutron Scattering Lengths and Cross Sections. *Neutron News* **1992**, *3* (3), 26-37.

(15) Salanne, M.; Simon, C.; Turq, P.; Madden, P. A. Calculation of Activities of Ions in Molten Salts with Potential Application to the Pyroprocessing of Nuclear Waste. *The Journal of Physical Chemistry B* **2008**, *112* (4), 1177-1183.

(16) Ohtori, N.; Salanne, M.; Madden, P. A. Calculations of the Thermal Conductivities of Ionic Materials by Simulation with Polarizable Interaction Potentials. *The Journal of Chemical Physics* **2009**, *130* (10), 104507.

(17) Desyatnik, V. N.; Katyshev, S. F.; Raspopin, S. P.; Chervinskii, Y. F. Density, Surface Tension, and Viscosity of Uranium Trichloride-Sodium Chloride Melts. *Soviet Atomic Energy* **1975**, *39* (1), 649-651.

(18) Perdew, J. P.; Burke, K.; Ernzerhof, M. Generalized Gradient Approximation Made Simple. *Physical Review Letters* **1996**, *77* (18), 3865-3868.

(19) Perdew, J. P.; Burke, K.; Ernzerhof, M. Perdew, Burke, and Ernzerhof Reply. *Physical Review Letters* **1998**, *80* (4), 891-891.

(20) Zhang, Y.; Yang, W. Comment on ``Generalized Gradient Approximation Made Simple''. *Physical Review Letters* **1998**, *80* (4), 890-890.

(21) Perdew, J. P.; Ruzsinszky, A.; Csonka, G. I.; Vydrov, O. A.; Scuseria, G. E.; Constantin, L. A.; Zhou, X.; Burke, K. Restoring the Density-Gradient Expansion for Exchange in Solids and Surfaces. *Physical Review Letters* **2008**, *100* (13), 136406.

(22) Grimme, S.; Antony, J.; Ehrlich, S.; Krieg, H. A Consistent and Accurate *ab initio* Parametrization of Density Functional Dispersion Correction (DFT-D) for the 94 Elements H-Pu. *The Journal of Chemical Physics* **2010**, *132* (15), 154104.

(23) VandeVondele, J.; Krack, M.; Mohamed, F.; Parrinello, M.; Chassaing, T.; Hutter, J. Quickstep: Fast and Accurate Density Functional Calculations Using a Mixed Gaussian and Plane Waves Approach. *Computer Physics Communications* **2005**, *167* (2), 103-128.

(24) Hutter, J.; Iannuzzi, M.; Schiffmann, F.; VandeVondele, J. CP2K: Atomistic Simulations of Condensed Matter Systems. *WIREs Computational Molecular Science* **2014**, *4* (1), 15-25.

(25) Kühne, T. D.; Iannuzzi, M.; Del Ben, M.; Rybkin, V. V.; Seewald, P.; Stein, F.; Laino, T.; Khaliullin, R. Z.; Schütt, O.; Schiffmann, F.; et al. CP2K: An Electronic Structure and Molecular Dynamics Software Package - Quickstep: Efficient and Accurate Electronic Structure Calculations. *The Journal of Chemical Physics* **2020**, *152* (19), 194103.

(26) VandeVondele, J.; Hutter, J. Gaussian Basis Sets for Accurate Calculations on Molecular Systems in Gas and Condensed Phases. *The Journal of Chemical Physics* **2007**, *127* (11), 114105.

(27) Goedecker, S.; Teter, M.; Hutter, J. Separable Dual-Space Gaussian Pseudopotentials. *Physical Review B* **1996**, *54* (3), 1703-1710. DOI: 10.1103/PhysRevB.54.1703.

(28) Nosé, S. A Unified Formulation of the Constant Temperature Molecular Dynamics Methods. *The Journal of Chemical Physics* **1984**, *81* (1), 511-519.

(29) *Debyer*; https://github.com/wojdyr/debyer

(30) Tucker, M. G.; Keen, D. A.; Dove, M. T.; Goodwin, A. L.; Hui, Q. RMCProfile: Reverse Monte Carlo for Polycrystalline Materials. *Journal of Physics: Condensed Matter* **2007**, *19* (33), 335218.

(31) Zhang, Y.; Eremenko, M.; Krayzman, V.; Tucker, M. G.; Levin, I. New Capabilities for Enhancement of *RMCProfile*: Instrumental Profiles with Arbitrary Peak Shapes for Structural Refinements Using the Reverse Monte Carlo Method. *Journal of Applied Crystallography* **2020**, *53* (6), 1509-1518.

(32) Silvi, B.; Savin, A. Classification of Chemical Bonds Based on Topological Analysis of Electron Localization Functions. *Nature* **1994**, *371* (6499), 683-686. DOI: 10.1038/371683a0.

(33) Becke, A. D.; Edgecombe, K. E. A Simple Measure of Electron Localization in Atomic and Molecular Systems. *The Journal of Chemical Physics* **1990**, *92* (9), 5397-5403.

(34) Adamo, C.; Barone, V. Toward Reliable Density Functional Methods Without Adjustable Parameters: The PBE0 Model. *The Journal of Chemical Physics* **1999**, *110* (13), 6158-6170.

(35) *Gaussian 16 Rev. A.03*; Wallingford, CT, 2016.

(36) Dunning, T. H., Jr. Gaussian Basis Sets for Use in Correlated Molecular Calculations. I. The Atoms Boron Through Neon and Hydrogen. *The Journal of Chemical Physics* **1989**, *90* (2), 1007-1023.

(37) Dolg, M.; Stoll, H.; Preuss, H.; Pitzer, R. M. Relativistic and Correlation Effects for Element 105 (Hahnium, Ha) A Comparative Study of M and MO (M = Nb, Ta, Ha) Using Energy-Adjusted *ab initio* Pseudopotentials. *Journal of Physical Chemistry* **1993**, *97* (22), 5852-5859.

(38) Glendening, E. D.; Landis, C. R.; Weinhold, F. Natural Bond Orbital Methods. *WIREs Computational Molecular Science* **2012**, *2* (1), 1-42.

(39) *NBO 7.0*; Theoretical Chemistry Institute, University of Wisconsin, Madison, 2018.

(40) Glendening, E. D.; Landis, C. R.; Weinhold, F. NBO 7.0: New Vistas in Localized and Delocalized Chemical Bonding Theory. *Journal of Computational Chemistry* **2019**, *40* (25), 2234-2241.

(41) Bader, R. F. W. Atoms in Molecules. *Accounts of Chemical Research* **1985**, *18* (1), 9-15.

(42) Lu, T.; Chen, F. Multiwfn: A Multifunctional Wavefunction Analyzer. *Journal of Computational Chemistry* **2012**, *33* (5), 580-592.

(43) Pettersen, E. F.; Goddard, T. D.; Huang, C. C.; Couch, G. S.; Greenblatt, D. M.; Meng, E. C.; Ferrin, T. E. UCSF Chimera—A Visualization System for Exploratory Research and Analysis. *Journal of Computational Chemistry* **2004**, *25* (13), 1605-1612.

(44) Kresse, G.; Furthmüller, J. Efficiency of ab-initio total energy calculations for metals and semiconductors using a plane-wave basis set. *Computational Materials Science* **1996**, *6* (1), 15-50.

(45) Kresse, G.; Hafner, J. Ab initio molecular dynamics for liquid metals. *Physical Review B* **1993**, *47* (1), 558-561.

(46) Anisimov, V. I.; Zaanen, J.; Andersen, O. K. Band theory and Mott insulators: Hubbard U instead of Stoner I. *Physical Review B* **1991**, *44* (3), 943-954. DOI: 10.1103/PhysRevB.44.943.

(47) Roy, S.; Sharma, S.; Karunaratne, W. V.; Wu, F.; Gakhar, R.; Maltsev, D. S.; Halstenberg, P.; Abeykoon, M.; Gill, S. K.; Zhang, Y.; et al. X-Ray Scattering Reveals Ion Clustering of Dilute Chromium Species in Molten Chloride Medium. *Chemical Science* **2021**, *12* (23), 8026-8035.

(48) Luzar, A.; Chandler, D. Hydrogen-Bond Kinetics in Liquid Water. *Nature* **1996**, *379* (6560), 55-57.

(49) Yu, X.; Sergentu, D.-C.; Feng, R.; Autschbach, J. Covalency of Trivalent Actinide Ions with Different Donor Ligands: Do Density Functional and Multiconfigurational Wavefunction Calculations Corroborate the Observed "Breaks"? *Inorganic Chemistry* **2021**, *60* (23), 17744-17757.
